# Impact of Primary Tumor Variables on Predicting Nodal Metastasis in Lower Extremity Marjolin’s Ulcer: A Retrospective Cohort Study

**DOI:** 10.7759/cureus.43673

**Published:** 2023-08-17

**Authors:** Deepak k Gupta, Parijat Suryavanshi, Gitika N Singh, Vijay Kumar, Shashi S Pawar, Vinod Jain

**Affiliations:** 1 General Surgery, King George's Medical University, Lucknow, IND; 2 Surgery, King George's Medical University, Lucknow, IND; 3 Surgical Oncology, King George's Medical University, Lucknow, IND; 4 Surgical Oncology, Indira Gandhi Institute of Medical Sciences, Patna, IND

**Keywords:** cutaneous squamous cell carcinoma (scc), nodal dissection, scar cancer, skin cancer, marjolin’s ulcer

## Abstract

Background

Marjolin's ulcer or scar carcinoma is a rare disease arising from the conversion of chronic scar into malignancy. Studies show Marjolin's ulcer squamous cell carcinoma has more chances of lymph nodal metastasis and is more aggressive with a worse survival rate. To date, no established guidelines exist for managing regional lymph nodes in cases of Marjolin's ulcer with clinically N0 nodes. Observation vs elective node dissection remains an option. In developing countries, long-term follow-up is not consistently leading to the risk of patients being kept on observation for regional nodes; presenting late with inoperable regional nodes is possible.

This study aims to identify clinicopathological factors of lower extremity Marjolin's ulcer, which are associated with a high risk of inguinal lymph node metastasis. Identifying such risk factors may help provide a rationale for performing elective nodal dissection instead of observation in high-risk cases.

Material and methods

All clinically N0 lower extremity Marjolin's ulcer cases, more than 3 cm in size, treated at King George's Medical University, India, during the last five years, have been included in this study. Demographic, clinical, and pathological data of eligible patients were retrieved from institutional records. Various clinical and pathological factors were studied and correlated with lymph node positivity, and the strength of the correlation was tested using statistical methods. Factors correlated strongly with inguinal lymph node positivity were identified as high-risk factors.

Results

A total of 66 patients with lower extremity Marjolin's ulcer had no preoperative pathologically confirmed inguinal lymph nodes documented by ultrasonography and fine needle aspiration cytology. All patients underwent surgery for primary, followed by elective, inguinal lymph nodal dissection. The majority were males (n=51/66; 71%), and the most common age group was 30-50 years (n=40/66; 60%). The leg was the most common site (n=31/66; 47%). The least common site was below the ankle (n=14/66; 22%). Maximum dimension ranged from 3 cm to >15 cm, with the majority between 6 and 10 cm (n=40/66; 56%). Extension beyond the scar site was present in 24% (n=15/66) of patients. Most of the lesions in this study were well differentiated, 85% (n=56/66), and moderately differentiated, 15% (n=10/66), and none of the lesions was poorly differentiated. Perineural invasion, lymphovascular invasion, tumor necrosis, and extension below subcutaneous tissue were present in 82%, 14%, 28%, and 26%, respectively. Of 66 patients, 21.2% (n=14/66) had pathological nodal disease after elective nodal dissection. Perineural invasion (p<0.0001), depth of lesion (p<0.0001), and tumor necrosis (p=0.0002) had a statistically significant correlation with node metastasis. On ROC curve analysis, 7.5 cm was the cut-off size, above which chances of nodal metastasis increased significantly.

Conclusions

Marjolin's ulcer patients with no preoperative positive nodes may be segregated into high-risk and low-risk groups as per their risk of harboring cancer cells in regional lymph nodes. Those having one or more of the following risk factors should be classified as high risk: dimension more than 7.5 cm, presence of perineural invasion, tumor necrosis, and deep tumors extending below subcutaneous tissue. We recommend that such patients undergo prophylactic regional lymph node dissection instead of observation during primary surgery.

## Introduction

Malignant transformation of chronic burn scars was known to humanity as early as 100 AD [1]. Later, several descriptions appeared in various reports about growths rising in chronic burn scars. One of the well-known descriptions was by Jean Nicholas Marjolin, a French surgeon, in 1828. Giving a befitting credit, Da Costa and Fordyce (1903) called these tumors arising from ulcers as "Marjolin's ulcer." Several reports of ulcers developing in chronic scars were reported [2]. Hence, they were called cicatricial scars or scar carcinomas. Marjolin's ulcer refers to any carcinoma arising from the chronic scar.

The exact incidence of Marjolin's ulcer is not well known, but it is accepted as a rare disease. The incidence of a chronic wound undergoing malignant transformation is nearly 1.8% to 2%, and the average age group of the fifth decade of life is the most common [3]. Several regional variations of these ulcers have been well reported, like kangri ulcers in Kashmiri Indians. "Kangri" is an earthen oven filled with coal. It was used to keep the body warm by keeping it in touch with the abdomen underneath gowns. Such practice led to repeated burn injuries and the subsequent development of cancer. Similar incidences have been reported in Japan in the form of “Kairo burn cancer."

Full-thickness burns, left to heal by secondary intention, are more prone to malignant transformation into Marjolin's ulcer [4]. It is likely a multifactorial process with a genetic and environmental component playing a role in its development. The age of onset of scar is found to be inversely proportional to the latency period between scar and cancer [5]. Occurrence of Marjolin's ulcer is most commonly seen in the lower extremities (55%-70%), followed by the head and neck (20-25%), upper extremities (10-12%), trunk, and other rarer locations, including toe, eyelid, and lips [6].

The most common histological variant of Marjolin’s ulcers is squamous cell carcinoma ([SCC] 75%), with the other being basal cell carcinoma and melanoma [7]. The various etiologies of Marjolin's ulcer are burn eschars, cellulitis, osteomyelitis, pressure sores, and traumatic wound. Cutaneous SCC has a relatively low rate of metastasis, usually ranging from 3% to 23%. However, those arising from invasive Marjolin's ulcers have a metastasis rate of 27.5%-40% [8]. The overall mortality from Marjolin’s is around 21% [9]. Regional nodal metastasis is associated with a worse prognosis. Controversy still exists on whether to perform elective nodal dissection in patients with clinical N0 nodes.

Due to the inconsistent follow-up of patients post-primary surgery, especially in developing countries, the patient might not present for routine follow-up for screening for lymph node metastasis; hence, if the preoperative clinicopathological features are suggestive of significantly high chances of inguinal lymph node metastasis, performing ipsilateral elective inguinal lymph node dissection along with the surgery of primary lesion makes sense as the patients might present at a late stage with inoperable regional lymph node metastasis post-primary surgery for Marjolin's ulcers.

At the very same time, lymph node dissection must be avoided in patients of Marjolin’s ulcer with a low risk of metastasis because lymph node dissections are associated with significant postoperative morbidity of lymphedema, surgical site infection, and flap necrosis, and the benefit of performing lymph node dissection in low-risk disease is questionable. There are no clear guidelines for performing an elective nodal dissection in the case of node-negative extremity Marjolin’s ulcer.

In order to reduce the number of prophylactic lymphadenectomies in patients with clinically negative regional lymph nodes, the pathological findings of the primary tumor can define the prognostic risk of lymph node metastasis, allowing the identification of patients at high risk. Extension of the primary tumor, histologic grade, growth pattern, lymphovascular invasion (LVI), and perineural invasion (PNI) are the histopathological variables assessed most frequently to predict regional lymph node involvement. The retrospective nature of the series, the need for more data on a large cohort of patients, and the single-center setting of the studies limit the reliability of published data.

This study assesses the clinicopathological features of the lower extremity of Marjolin's ulcer, which are associated with increased chances of nodal metastasis, to draw a rationale for prophylactic inguinal nodal dissection in Marjolin’s ulcer.

## Materials and methods

Study design and population

This study is a retrospective study of biopsy-proven SCC arising from chronic scars seen in King George's Medical University, Lucknow, Uttar Pradesh, India, over five years (2017-2022). Marjolin's ulcers, less than 3 cm, were excluded from this study due to the low probability of nodal metastasis. Those patients with no nodal dissection performed were excluded from this study. The details of all included patients were retrieved from institutional records, and their demographic, clinical, radiological, and histopathological data were recorded.

In each patient, the following clinical variables were evaluated: lesion site (proximal or distal), tumor dimension, extension beyond the scar, and clinically palpable nodes. Tumors of the lower extremities were classified into three groups based on the lesion site: ankle-foot, knee-leg, and thigh. If more than one site is involved, then the main epicenter is taken as the site of the disease. The tumor dimension is the maximum dimension of a tumor measured clinically in centimeters in any plane. Extension beyond the scar site was observed by clinical examination. Clinical lymph node status was assigned by inguinal lymph node palpation and high-resolution ultrasonography (USG) of the inguinal area. Enlarged nodes underwent USG-guided fine needle aspiration cytology. Preoperative positive nodal disease was excluded from this study. Only preoperative node-negative cases were assessed.

The following pathological variables of the primary tumor were assessed: tumor differentiation, LVI, PNI, tumor necrosis, and depth of lesion. Tumor differentiation was based on Broder’s classification: grade 1, well-differentiated; grade 2, moderately differentiated; and grade 3, poorly differentiated. LVI was defined as tumor emboli within endothelium-lined spaces. PNI was defined as tumor invasion in, around, and through the nerves.

Ethical approval

Ethical approval to conduct this study was taken from the institutional ethical committee.

Statistical analysis

Data were analyzed using statistical software SPSS Version 25 (IBM Corp., Armonk, NY, USA). The continuous variables were evaluated by mean (standard deviation) or range value when required. The dichotomous variables were presented in number/frequency and were analyzed using the chi-square test. For comparison of the means between the two groups, analysis by Student t-test was used. Correlation analysis was conducted using the Spearman r test. The cutoff value was identified using receiver operator characteristics (ROC) analysis. All the analysis was done at a 95% confidence interval, and a p-value of <0.05 was considered significant.

## Results

During this study period, 66 patients with lower extremity Marjolin's ulcer were identified who were treated and were clinically N0. The number of male patients was 51 (77%), and female patients were 15 (23%). The majority of patients were in the age group of 30-50 years, with 40 patients (60%), followed by 22 patients above 50 years (34 %), and a small percentage of less than 30 years of age, four patients (6%). Thirty-one (47%) patients had lesions between the knee and ankle, 21 (31%) patients had above-knee lesions, and the rest 14 (22%) patients had distal below-ankle lesions (Table 1).

**Table 1:** Occurrence of various clinical and pathological features in cases

Clinical and pathological variables	Incidence (n = 66)
Gender	
Male	51 (77%)
Female	15 (23%)
Age	
<30 years	04 (6%)
30-50 years	40 (60%)
>50 years	22 (34%)
Site	
Thigh	21 (31%)
Leg	31 (47%)
Ankle	14 (22%)
Dimension	
3-5 cm	14 (22%)
6-10 cm	40 (56%)
11-15 cm	10 (15%)
>15 cm	02 (7%)
Differentiation	
Well differentiated	56 (85%)
Moderately differentiated	10 (15%)
Poorly differentiated	None
Perineural invasion	
Present	54 (82%)
Absent	12 (18%)
Lymphovascular invasion	
Present	09 (14%)
Absent	57 (86%)
Tumor necrosis	
Present	18 (28%)
Absent	48 (72%)
Palpable nodes	
Present	49 (74%)
Absent	17 (26%)
Depth of lesion	
Upto subcutaneous tissue	49 (74%)
Below subcutaneous tissue	17 (26%)

The maximum dimension of the lesion ranged from 3 cm to >15 cm. The majority of patients (40; 56%) had dimensions between 6 and 10 cm, 14 (22%) patients had dimensions between 3 and 5 cm, 10 (15%) patients had dimensions between 11 and 15 cm, and rest two patients had dimensions >15 cm. In 24% of cases, the disease extended beyond the scar site onto normal adjacent skin. Well-differentiated cancer was most common in 56/66 cases (85%). The rest were moderately differentiated. PNI was present in 82% (54/66) patients. LVI was present in 14% of cases. Tumor necrosis was present in 18/66 (28%) patients. Depth of lesion was classified into two groups: disease extending up to subcutaneous tissue and disease extending deep to it. In 26% of cases (17/66), the disease extended below the subcutaneous plane. Clinically palpable lymph nodes were present in 74 % (49/66). In a histopathological study, 14 patients had confirmed pathologically positive nodes (21.2%) (Table 1).

Various parameters, such as site of the lesion (distal or proximal extremity), maximum dimension in cm, extension beyond scar, tumor differentiation, PNI, LVI, depth of lesion, clinically palpable nodes, and tumor necrosis, were studied and correlated with pathological lymph node positivity (Tables 2, 3). There was no statistically significant correlation of clinical parameters with lymph node involvement, such as age (p=0.89), site of disease (p=0.3), maximum dimension (p=0.4), extension beyond scar (p=0.4), and clinically palpable nodes (p=0.6).

**Table 2:** Clinical variables predictive of lymph node involvement: univariate analysis (Pearson chi-square test) in the entire cohort of patients

Lymph node metastasis vs. clinical parameters	Spearman r	95% confidence interval	P-value
Site of lesion	-0.1061	-0.3459 to 0.1466	0.3964
Maximum dimension (cm)	0.08487	-0.1676 to 0.3269	0.498
Outside extension	0.09376	-0.1588 to 0.3348	0.454
Clinically palpable nodes	0.05137	-0.2001 to 0.2965	0.6821

**Table 3:** Pathological variables predictive of lymph node involvement: univariate analysis (Pearson chi-square test) in the entire cohort of patients *Indicates statistically significant p-values.

Lymph node metastasis vs. pathological parameters	Spearman r	95% confidence interval	P-value
Differentiation	0.09084	-0.1617 to 0.3322	0.4682
Perineural invasion	0.6638	0.5689 to 0.8192	<0.0001*
Lymphovascular invasion	0.2258	-0.02445 to 0.4494	0.0683
Depth of lesion	0.6267	0.4476 to 0.7574	<0.0001*
Tumor necrosis	0.4413	0.2161 to 0.6219	0.0002*

Among pathological parameters, PNI in the tumor had a statistically significant correlation with lymph node involvement (p<0.0001). Depth of lesion (p <0.0001) and presence of tumor necrosis (p 0.0002) also had a statistically significant correlation with lymph node involvement. There was a non-significant trend toward a correlation between LVI and lymph node involvement (p=0.06). There was no statistically significant correlation of tumor differentiation with nodal metastasis (p=0.4).

**Figure 1:**
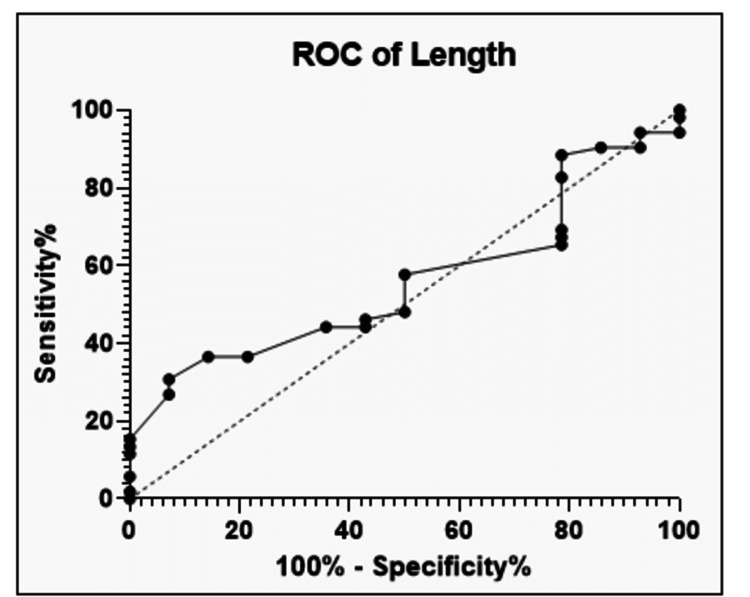
ROC curve analysis for the maximum dimension of the tumor ROC, receiver operator characteristics

ROC curve analysis was conducted, and the 7.5-cm maximum dimension was the cutoff, above which there was a significant increase in the risk of lymph node involvement (Figure 1). However, the maximum dimension of the lesion did not have a statistically significant correlation with nodal metastasis on the Spearman r test (p=0.4).

## Discussion

Marjolin's ulcer is a relatively rare disease. Hence, the higher number of cases in our series can be due to the following contributory factors: our institute is one the most prominent tertiary referral center of Uttar Pradesh, which is India's most populous state. It also receives patients from neighboring states. Most patients attending our institute are from lower socio-economic strata. Marjolin's ulcer is more common in lower socio-economic strata due to increased incidence of predisposing factors such as cellulitis, burn, and trauma, and there is a delay in timely diagnosis and referral. Most of these patients had their wounds managed as non-healing wounds for weeks to months before referral. Most cases occurred in the age group of 30-50 years. Reasons may be due to more incidence of chronic non-healing wounds and scars in this gender as they are more prone to cellulitis and trauma. The male-to-female incidence and age of diagnosis observed in the present study were similar to other studies on Marjolin's ulcer [10]. The mean latency period, age of injury causing scar, and mode of initial injury could not be reliably assessed due to inconsistent data recording and hence are not mentioned in our study. Marjolin's ulcers of non-extremity areas were excluded from our study due to less number, and in the majority, nodal dissection was not done. Moreover, studies have shown that Marjolin's extremity ulcer has a higher chance of metastasis; therefore, prophylactic lymph node dissection seems more reasonable in these cases [2].

Most of Marjolin's ulcers in the present study were well differentiated among pathological characteristics. This finding concurs with other studies showing that poorly differentiated tumors are less common in Marjolin's ulcers than non-Marjolin's SCC [11]. Studies evaluating the survival outcomes of Marjolin's ulcer and non-marjolin's SCC are minimal due to the rarity of the disease. Limited series have shown Marjolin's ulcer to have a worse prognosis than non-Marjolin's SCC despite having better tumor differentiation. This contradictory finding emphasizes the importance of other factors in Marjolin's ulcer contributing to its aggressive behavior, as in a study by Iqbal et al. [12]. PNI and LVI are risk factors associated with increased chances of lymph node metastasis and worse outcome in SCC at several sites, including skin cancer, penile cancer, oral cancer, and anal cancer. In the present study, PNI was present in most patients.

However, there was a low incidence of LVI. The reason for this low expression of LVI in tumors in our study is unclear. Most cases have disease limited to subcutaneous tissue, probably because the initial injury and scar were limited to superficial layers. Lymph nodes were palpable in most cases; however, fewer had pathologically positive nodes (21.2%). The rate of lymph node metastasis reported from several studies has been variable, with some studies having a rate of metastasis as high as 60%. Most studies quote nodal metastasis rates ranging from 20% to 40%. Higher nodal metastatic rate is thought to be due to the permeation of the thick scars by tumor tissue for a long time; therefore, immune systems control over the tumor is not present [13]. Finally, the heavily mutated tumor rapidly spreads to nodes when it penetrates eschar. Most lesions in the present study were between 6 and 10 cm, and in around one-third of cases, ulcers extended beyond the margins of the scar onto normal skin. This extension beyond the scar is considered a high-risk factor as the tumor accesses normal lymphatics in healthy tissue.

Inguinal lymph node dissection carries significant morbidity in flap necrosis, seroma, lymphorrhea, and, most significantly, lymphedema. Several types of research in penile cancer and skin cancer have focused on predicting lymph node metastasis by using tumor clinicopathological variables [8]. Sentinel lymph node biopsy is now becoming the norm in several cancers. This study has been an attempt to avoid lymph node dissection in cases having low chances of metastasis and, thereby, its morbidity [14]. Long-term morbidity puts heavy financial and emotional burdens on patients and stretches the health system. In developing countries like India, the majority population is from rural backgrounds and lower socio-economic status. Avoiding long-term morbidity of nodal dissection means avoiding a heavy financial burden on the already stretched finances of poor patients. With this background, this study was planned to identify high-risk factors for lymph node metastasis in Marjolin’s ulcer by assessing various Clinical and pathological tumor variables and their correlation with the incidence of lymph node metastasis. Site of the lesion (distal or proximal extremity), maximum dimension in cm, extension beyond scar, tumor differentiation, PNI, LVI, depth of the lesion, clinically palpable nodes, and tumor necrosis were studied and correlated with pathological lymph node positivity by using Pearson correlation- univariate analysis. None of the four clinical parameters assessed in our study had any significant correlation with nodal metastasis. Similar findings have been reported from other studies, where the dimension of the lesion did not correlate with nodal metastasis [15]. Extension beyond the scar was expected as a high-risk factor for lymphatic metastasis, but no significant correlation was present. We postulate this to be due to a low number of patients with extension beyond scar in the present study. Taking the scar in a three-dimensional view, as the lesion goes deep, it may cross the scar in a vertical aspect. In a sense, evaluating the depth of the lesion of the tumor, as we did in the present study, can be taken as a surrogate marker for extension beyond the scar in the vertical aspect.

Clinically palpable lymph nodes had little correlation with nodal positivity. This finding is because such ulcers are infected with necrotic debris getting infected and deep crevices prevent adequate cleaning. A similar result is reported from other studies on Marjolin's ulcer, skin cancer, and penile cancer, whereby clinical lymph node presence had little correlation with pathological involvement due to the high incidence of enlargement secondary to infection [16]. Among pathological parameters, PNI, tumor necrosis, and depth of lesion had statistically significant correlations with the incidence of nodal metastasis in the present study. PNI can exist independently of LVI, as in the present study, and it may be the sole metastasis method for some tumors. PNI occurs in several malignancies, such as pancreatic, gastric, colorectal, prostate, oral, and cervical cancer. It is an essential factor influencing malignant tumor pathological characteristics and prognosis, associated with decreased survival. Tumor necrosis is a well-known feature of many aggressive malignancies and portends a poor prognosis. Tumor necrosis increases the chances of dissemination of tumor cells; however, the mechanism is unclear. A recent study has shown increased angiopoietin-like 7 (Angptl-7) expression in the peri necrotic tumoral zone. Angptl-7 is known to increase vascular permeability and dissemination [17]. Depth of lesions is an essential factor influencing the risk of lymph node metastasis in several cancers such as melanoma, skin cancer, oral cancer, and cervical cancer.

As the depth of the tumor increases, the chances of lymphatic metastasis increase as tumor cells gain access to new lymphatic channels once it crosses the scar in deeper planes. It is like riding a rough countryside road, and a highway suddenly comes. Measuring the depth of the tumor can be done in two ways: by using anatomical layers as a reference and measuring tumor depth accurately in millimeters. In the present study, since we have tried to correlate preoperative tumor factors, which can predict lymphatic metastasis, we have adopted the first method, which can be evaluated by preoperative imaging. The correlation of LVI with nodal metastasis showed a non-significant trend. This fact may be attributed to the low incidence of LVI in our series, thereby precluding any meaningful correlation. However, it is a well-recognized adverse risk factor for nodal metastasis and survival.

Although the maximum dimension of the lesion had no significant correlation with nodal metastasis, similarly reported in other studies, we evaluated the dimension of the lesion factor to find a cutoff size above which chances of nodal metastasis may increase significantly. ROC curve analysis showed a cutoff of 7.5-cm maximum dimension of lesion, above which chances of nodal metastasis increase.

The present study helps decide whether clinically node-negative cases of Marjolin's ulcer should undergo prophylactic regional lymph node dissection. We have taken the first step in fulfilling this aim by identifying tumor variables that can be measured or seen in preoperative clinical and imaging workups to identify high-risk features that portend a higher chance of nodal metastasis. Regional lymph node dissection may be justifiable in such cases.

The main limitation of this study is its retrospective nature. However, conducting a prospective study on rare diseases is often not feasible due to the rarity of the disease, as the target number may be unattainable. On the other hand, retrospective studies provide important extensive data and information regarding a rare disease. The smaller sample size is because Marjolin's ulcer is a rare disease.

## Conclusions

Marjolin's ulcer patients with no preoperative positive nodes may be segregated into high-risk and low-risk groups as per their risk of harboring cancer cells in regional lymph nodes. Those having one or more of the following risk factors should be classified as high risk: dimension more than 7.5 cm, presence of PNI, tumor necrosis, and deep tumors extending below subcutaneous tissue. We recommend that such patients undergo prophylactic regional lymph node dissection instead of observation during primary surgery.
